# Prenatal diagnosis of a rare variant of harlequin ichthyosis with literature review

**DOI:** 10.1186/s12880-021-00586-4

**Published:** 2021-03-21

**Authors:** Yi Zhou, Liang Li, Ling Wang, Chaoxue Zhang

**Affiliations:** Department of Ultrasound, First Affiliated Hospital of Anhui Medical University, 218 Jixi Road, Hefei, 230022 Anhui China

**Keywords:** Prenatal, Harlequin ichthyosis, ABA12, Congenital, Ultrasound

## Abstract

**Background:**

Harlequin ichthyosis (HI) is a rare and severe genetic skin disorder that occurs within the developing foetus. Due to the extremely poor prognosis, prenatal diagnosis becomes very important, especially for foetuses with no family history. There are few reports on prenatal diagnosis in PubMed.

**Case presentation:**

We report two cases of HI with no family history who were diagnosed by prenatal ultrasound. We searched for reports on the prenatal ultrasonic diagnosis of HI over nearly two decades and summarized the sonographic features of HI, the reasons for missed diagnoses and matters needing attention. A total of 10 articles of congenital harlequin ichthyosis diagnosed by prenatal ultrasound in PubMed were retrieved. There have been even fewer reports of late-trimester disease with no family history. Combining the two cases we reported with the literature review, we summarize the ultrasonic image characteristics of HI.

**Conclusion:**

HI can be easily detected by 2D ultrasound combined with 3D, but attention should be paid to a systematic examination in the third trimester of pregnancy according to the clinical characteristics of the disease.

## Background

Harlequin ichthyosis (HI) is a rare and severe genetic skin disorder that occurs within the developing foetus. Also known as heavy colloidal foetus or ugly foetus, its incidence is very low, ranging from 1/300 000 to 1/1 000 000 [1]. HI typically exhibits large, thick, plate-like scales covering the whole body and is associated with severe ectropion, eclabium and flattened ears. It later develops into a severe scaling erythroderma. The probability of mortality for infants with HI is high due to respiratory failure, loss of fluid or skin infections, and there are no effective treatments [2]. As such, prenatal diagnosis of foetal HI is critical for appropriate perinatal and postnatal management and to prepare parents for future pregnancies. Prenatal diagnosis mainly relies on ultrasonic, amniotic fluid and umbilical cord blood molecular detection. However, due to the timing and characteristics of its onset, most of the clinical phenotypes have passed the optimal examination time, making prenatal diagnosis more difficult, especially in cases without a family history. Only a few cases are reported in PubMed. We report the prenatal diagnosis of a harlequin foetus based only on ultrasound in two cases without a family history. The diagnostic value of prenatal ultrasound in congenital harlequin ichthyosis was also summarized.

## Case presentation

Case 1: A 31-year-old pregnant woman, gravida 3, para 0. The first two pregnancies were spontaneous abortions in the early stages of pregnancy. The eighth week of this pregnancy was marked by vaginal bleeding and abdominal pain, and a heparin injection was given to prevent pregnancy loss. All examinations were conducted in our centre. The results of the 11-week early pregnancy screening and the 21-week middle pregnancy screening were normal. A routine prenatal examination was normal at 27 weeks.

The woman underwent genetic tests at sixteen weeks gestation for Down's syndrome, neural tube defects and trisomy 18 syndrome, the results of which were all negative. A karyotype analysis of the amniotic fluid at twenty weeks of gestation exhibited no chromosomal abnormalities. Both husband and wife had no history of disease or family history, and both had a normal chromosome examination. Early viral infection was denied.

**Fig. 1:**
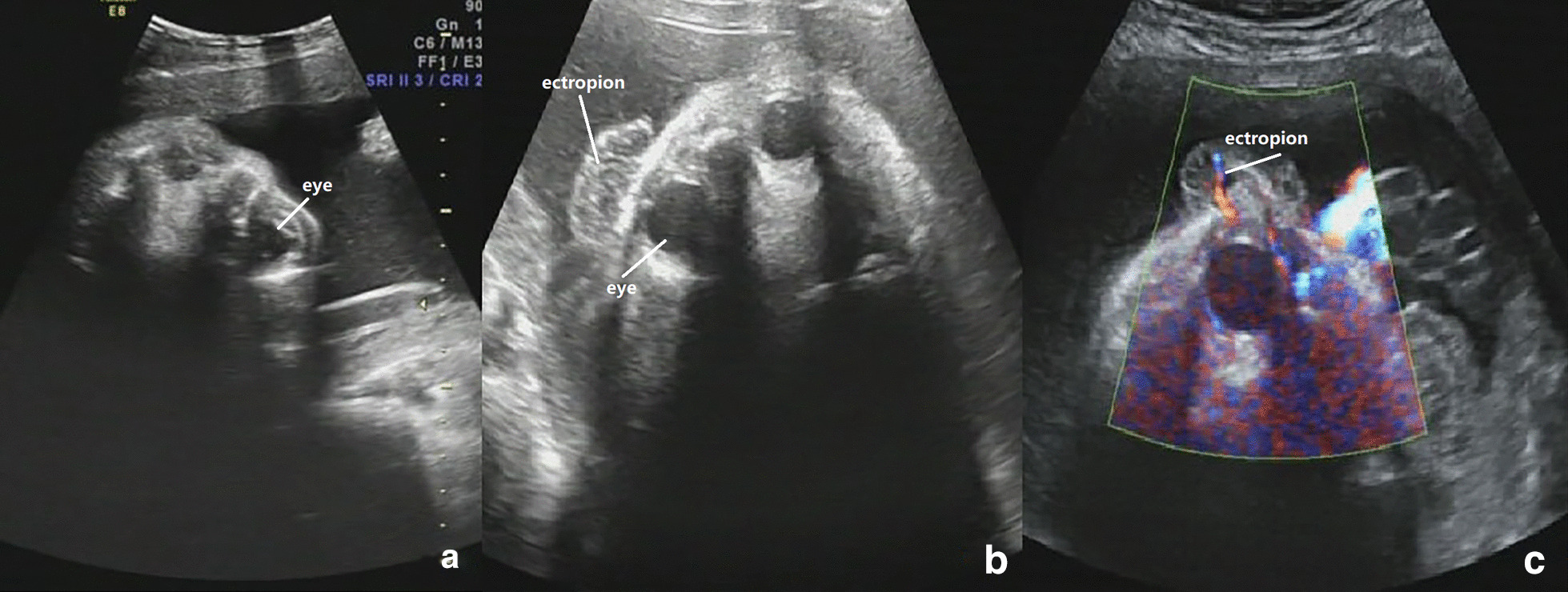
**a** 2-D ultrasound: no abnormality was observed in both eyes of the fetus at 27 weeks. **b** 2-D ultrasound: fetal ectropion at 31 weeks. **c** Color doppler display: blood flow signals from the eyes are detected in the everted eyelid

**Fig. 2:**
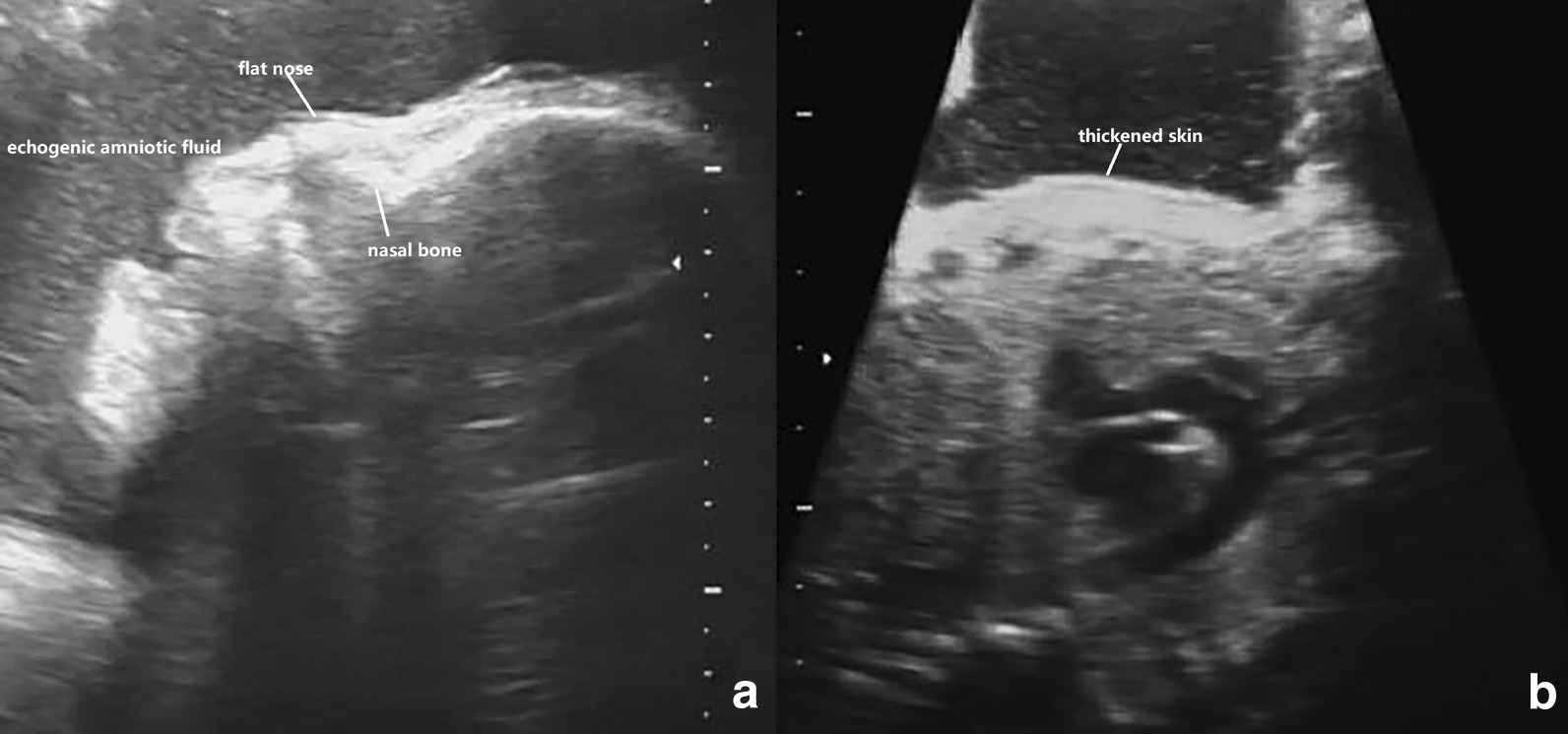
**a** 2-D ultrasound: flat nose and echogenic amniotic fluid at 31 weeks. **b** 2-D ultrasound: the long axis of the body shows locally thickened skin with a clear boundary with normal skin at 31 weeks

**Fig. 3:**
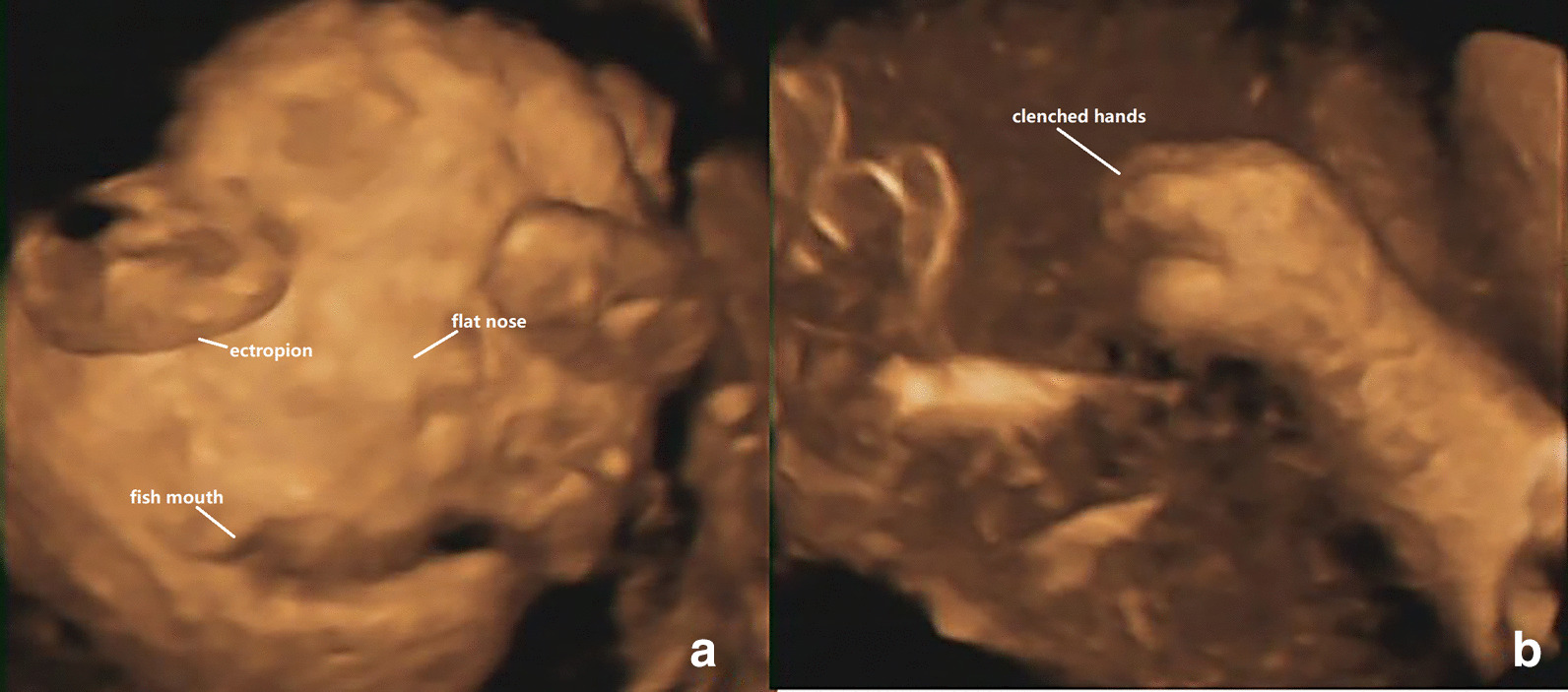
**a** 3-D ultrasound: visual display of abnormal fetal face, include: ectropion, the nose flat. The mouth could not be closed and was like a fish's mouth. **b** 3-D ultrasound: excessive flexion of fingers

Typical sonogram features were observed in the 31-week late pregnancy exam, as follows: thickening of the skin, ectropion, a short nasal bone, a flat nose, a mouth that could not close and that was similar to a fish's mouth; and excessive flexion of the fingers and toes. Foetal movement was not obvious during the examination, and there was echogenic amniotic fluid (Figs. 1, 2, 3). After communicating with the pregnant couple, the decision was made to terminate the pregnancy.

**Fig. 4:**
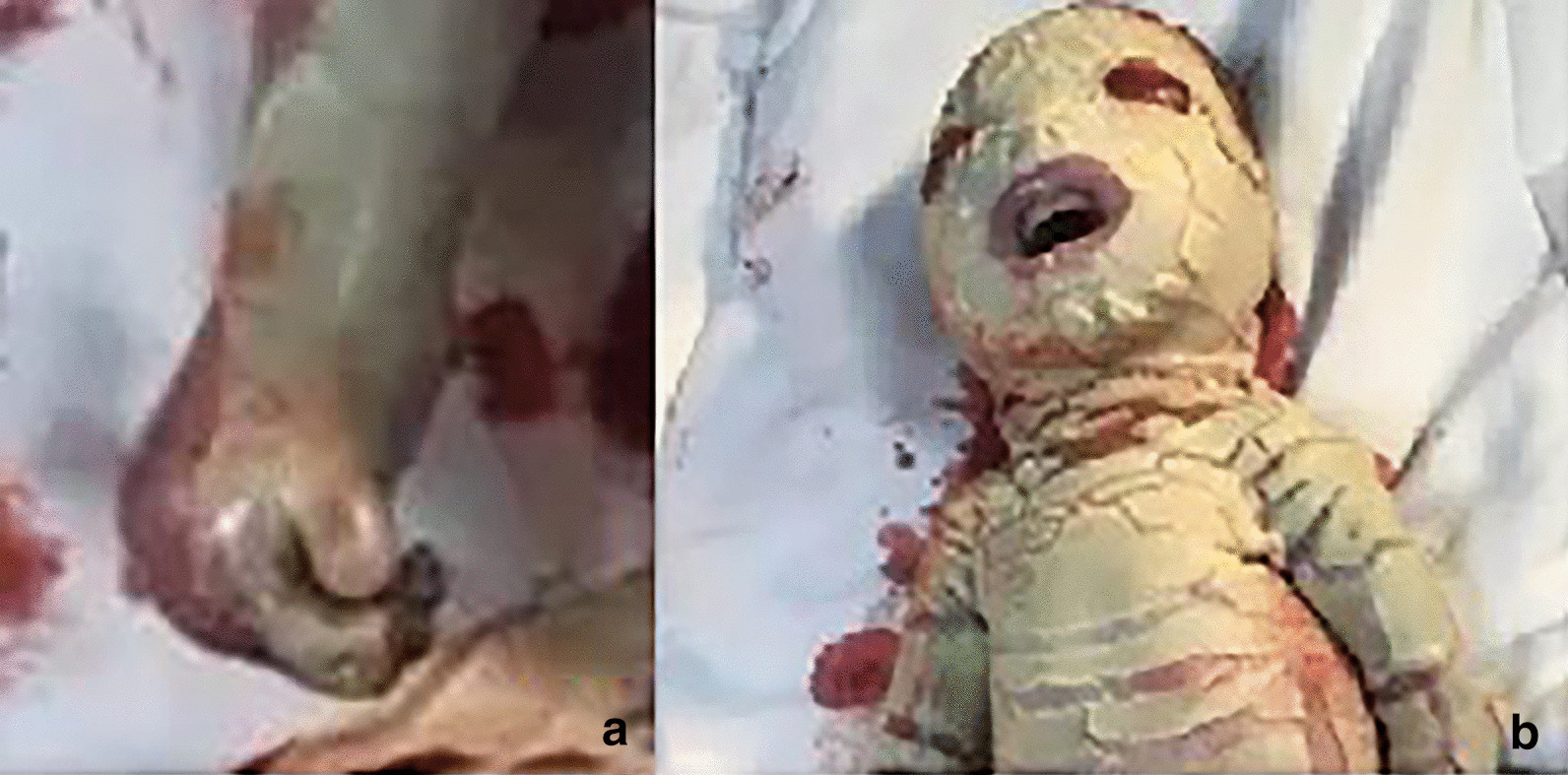
**a** Gross specimen display: excessive flexion of fingers. **b** The gross specimen was consistent with the prenatal ultrasound diagnosis

The gross specimen after induced labour had the following main phenotypic features: dry, scaly, fish-like skin consisting of hyperkeratosis with deep erythematous fissures between thick yellowish armour-like plaques involving the entire body surface; the eversion of the superior and inferior eyelids; a fish mouth appearance; the absence of eyelashes and eyebrows; sparse hair; abnormal flattened ears; a broadened flat nose; microcephaly; abnormally fixed limbs and fingers, and toes in rigid flexion (mitten-like hands) due to the inability of the skin to expand (Fig. 4). Specimens for histopathological analyses demonstrated epidermolytic hyperkeratosis ichthyosis and confirmed the diagnosis of HI.

Case 2: A 28-year-old pregnant woman, gestational age 30 weeks, G1 P0. During the prenatal examination, ultrasound at other hospitals suggested that the foetus had abnormal facial features, so the case was transferred to our centre. Both the husband and wife had no history of disease or family history, and both had a normal chromosome examination. Nuchal translucency (NT) and the foetal examination at 18–24 weeks showed no abnormality at other hospitals. Non-invasive prenatal testing showed no abnormalities. The pregnant woman recently became aware of abdominal enlargement and less foetal movement and suspected her amniotic fluid had increased, so she went to the hospital.

Ultrasound examination at our hospital showed that the biological values of the foetus were significantly lower than the actual gestational age, the equivalent of 24 w. Other sonographic findings were similar to case 1. After communicating with the family, the decision was made to terminate the pregnancy. After induction of labour, the gross specimen was consistent with the prenatal ultrasound. It was confirmed HI.

Relevant reports on prenatal ultrasound of congenital harlequin ichthyosis over nearly 20 years were searched in PubMed, and their sonographic characteristics were summarized and analysed.Combined with the literature reports, the prenatal ultrasonic signs and values of congenital harlequin ichthyosis caused by the ABA12 mutation will be discussed. A total of 10 articles of congenital harlequin ichthyosis diagnosed by prenatal ultrasound were retrieved from PubMed. There have been even fewer reports of late-trimester disease with no family history. This paper analyses the literature reports within 20 years, combined with the ultrasonic sonographic features of two cases of harlequin scale disease reported in this study, and analyses its ultrasonic signs.

Sonographic findings of 2D ultrasound combined with 3D imaging are as follows: The local skin of the body is thickened like armor, and there is a clear boundary with the normal skin, and the skin line echo is uneven and stratified.The nasal bones are shorter, the nose is flattened,

Keep the mouth open like a fish,The fingers and toes were in a fixed flexion position due to the abnormal pulling of the skin, and no significant changes were observed during scanning.The shedding of the epidermis causes the amniotic fluid to present a cloudy sound image. Three-dimensional ultrasound can visualize these foetal abnormalities, and two-dimensional ultrasound can assist in the diagnosis. In addition, three-dimensional ultrasound can visually show the foetal abnormalities to the parents (Table 1, Figs. 1, 2, 3, 4).

**Table 1:** Comparison of the ultrasound findings of published cases of prenatal diagnosis in the last twenty years

Ultrasound findings	This case 1	This case 2	Liang et al. [7]	Pedro et al. [8]	Wei Jian et al. [9]	Tourette et al. [10]	Hazuku et al. [11]	Basgul et al. [12]	Kudla et al. [6]	Suresh et al. [13]	Vohra et al. [14]	Bongain et al. [15]
Polyhydramnis		** + **							** + **	** + **	** + **	
Echogenic amniotic fluid	** + **	** + **		** + **		** + **					** + **	
Peeling skin	** + **	** + **	** + **	** + **	** + **			** + **		** + **	** + **	
Subcutaneous edema				** + **								
Short umbilical cord									** + **			
Fetal growth restrictriction		** + **		** + **		** + **			** + **	** + **		** + **
microcephaly				** + **								** + **
Ectropion	** + **	** + **	** + **	** + **	** + **	** + **	** + **	** + **	** + **	** + **		
Flat nose	** + **	** + **	** + **	** + **	** + **	** + **		** + **	** + **		** + **	** + **
Macroglosssia				** + **				** + **			** + **	
Micrognathia				** + **					** + **			
Fish mouth	** + **		** + **	** + **								
Clenched hands and clubfeet	** + **		** + **	** + **	** + **	** + **			** + **	** + **	** + **	** + **
Abnormal ears	** + **		** + **	** + **	** + **			** + **	** + **			

## Discussion and conclusion

HI, otherwise known as keratosis diffusa foetalis, is the most severe and devastating form of autosomal recessive congenital ichthyoses. It is due to mutations in the ABC transporter ABCA12, a cell membrane transporter associated with lipid transportation. Foetuses carrying this mutation have defective lipid secretion within epidermal keratinocytes, leading to a loss of the skin lipid barrier and to the development of harlequin ichthyosis [3]. The incidence of the disease is very low, and effective treatment is lacking [4]. The probability of mortality for infants with HI is high, their prognosis is poor, and most neonates die shortly after delivery due to infection, heat loss, dehydration, electrolytic disturbances, or respiratory distress.

The risk of pregnancy recurrence is 25%, so prenatal diagnosis is important. In patients with family histories, fetoscopy with skin biopsy and ultrastructural examination of the hair canal and amniotic fluid cells may be helpful [5]. For those without a family history, many cases are missed.

We analysed the causes, and it is mainly because keratinization does not begin until 22 to 24 weeks of gestation in the normal foetus. After the discovery of the ABCA12 mutation, the diagnosis may be confirmed by chorionic villus or amniotic fluid samples [6], but in patients without a family history, the target gene is not routinely tested. Most of the characteristic features of ultrasound will only become apparent later in pregnancy when restricted skin development becomes a limitation to foetal growth and movements. During this gestational period, it is difficult to complete a systematic foetal examination, coupled with the pregnant woman's conceptual problems, and some foetuses are not examined during the third trimester, so a prenatal diagnosis is easily missed.In PubMed literature search, We found that in the past two decades, only a small number of cases worldwide have been definitively diagnosed by prenatal ultrasound. Of the only 10 reported fetuses, 5 pregnant women had an adverse pregnancy history, of which 1 was a clear family history.Only 3 cases developed in the second trimester, and the remaining 7 cases all developed in the third trimester.Most of the families decided to terminate the pregnancy after communication and prognosis evaluation, only 6 cases gave birth naturally, and no one has been reported alive so far,Therefore, early diagnosis should be possible.

In this study, the ultrasonic characteristics of HI were analysed by combining this case with previous literature reports, and they consist of (1) the presence of ectropion; (2) the presence of an abnormal double auricle; (3) the ridge of the nose is flat; (4)thickened skin can be seen on parts of the skin, which looks like armour; (5) double lip thickened valgus, a sustained state of an open mouth, like a fish’s mouth, is present; (6) limb contracture flexion fixation is present; (7) there is no obvious foetal movement during the examination; and (8) particles can be seen floating in the amniotic fluid. HI and Neu‐Laxova syndrome (NLS) have overlapping features, but each has the unique ultrasonic signs, so the differential diagnosis is not difficult. Eclabium, lack of microcephaly, and lack of oedema are typical of HI, whereas cataracts and a short umbilical cord are characteristics of NLS [5].

Three-dimensional ultrasound can assist in two-dimensional ultrasonic diagnosis by visually displaying foetal abnormalities, and the obtained images have photograph-like realism and allow the parents to understand the exact nature of the anomaly. A new step forward with 3D imaging appears to have been made in the case of the harlequin foetus; we have found that 3D imaging is essential to understand the 2D images and enable diagnosis.But three-dimensional ultrasound can't replace two-dimensional ultrasound,Most cases occur in late pregnancy.At this stage, there is less amniotic fluid and the fetal position is fixed. Most of the fetuses cannot complete three-dimensional imaging, and still need to complete diagnosis on a two-dimensional basis.

In conclusion, HI can be easily detected by 2D ultrasound combined with 3D ultrasound, but attention should be paid to a systematic examination in the third trimester of pregnancy according to the clinical characteristics of the disease to avoid missing a diagnosis of HI, especially in cases without a family history.

## Data Availability

The datasets used and/or analysed during the current study are available from the corresponding author on reasonable request.

## Abbreviations

HI: Harlequin ichthyosis
2D/3D: 2 Dimensional/3 dimensional
NT: Nuchal translucency
NLS: Neu‐Laxova syndrome
